# Bioactive peptides targeting dyslipidemia and atherosclerosis: from computational discovery and data bottlenecks to precision medicine translation

**DOI:** 10.3389/fmolb.2026.1818173

**Published:** 2026-05-21

**Authors:** Wei Yin, Qiang Wang, ZhiMing Zeng, Xu Xie

**Affiliations:** 1 Guangxi International Zhuang Medicine Hospital Affiliated to Guangxi University of Chinese Medicine, Nanning, Guangxi, China; 2 The Reproductive Hospital of Guangxi Zhuang Autonoumous Region, Nanning, Guangxi, China

**Keywords:** atherosclerosis, bioactive peptides, computational peptide discovery, dyslipidemia, lipid metabolism, machine learning, PCSK9 inhibitors, peptide therapeutics

## Abstract

Bioactive peptides have emerged as a promising therapeutic class for dyslipidemia and residual cardiovascular risk, operating through mechanisms that include apolipoprotein mimicry, PCSK9 inhibition, and modulation of cholesterol absorption and inflammation, yet translational success remains constrained by poor pharmacokinetics, limited oral bioavailability, and inconsistent clinical outcomes. Computational approaches, ranging from machine learning classifiers and protein language models to structure-guided docking and generative design, now accelerate peptide discovery by enabling high-throughput screening, rational optimization of stability and affinity, and *de novo* generation of function-tailored sequences. However, the field faces persistent bottlenecks: lipid-annotated peptide datasets are scarce and fragmented, negative sampling biases inflate model performance, and most predictive algorithms trained on antimicrobial or general bioactive peptide data generalize poorly to cardiovascular applications, with many computationally derived leads lacking orthogonal peptidomics or proteolytic validation and consequently yielding high false-positive rates. Recent successes, particularly the development of orally bioavailable macrocyclic PCSK9 inhibitors, demonstrate that integrated pipelines combining structure-based design, display technologies, and multi-parameter optimization can overcome these barriers. Moving forward, progress will require concerted efforts to build high-quality, standardized lipid-peptide repositories, adopt uncertainty-aware and domain-adaptive modeling strategies, and embed early-stage developability filters into computational workflows. The convergence of generative artificial intelligence, population genomics, and precision medicine may ultimately enable patient-tailored peptide therapeutics capable of addressing the heterogeneous nature of dyslipidemia and atherosclerotic disease.

## 1 Introduction

### 1.1 Dyslipidemia and cardiovascular diseases: clinical burden and unmet needs

Dyslipidemia remains one of the most prevalent modifiable risk factors for atherosclerotic cardiovascular disease worldwide. In a recent systematic review and meta-analysis including 206 population studies, the global prevalence of hypercholesterolemia was 24.1% and high LDL-C was 18.93%, while hypertriglyceridemia and low HDL-C remained highly prevalent at 28.8% and 38.4%, respectively. In parallel, a GBD analysis showed that the absolute annual deaths and DALYs attributable to high LDL-C increased by 46% and 41% from 1990 to 2019, underscoring the persistent unmet clinical need for new lipid-lowering strategies (Ballena-Caicedo et al., 2025; Du et al., 2022).

### 1.2 Bioactive peptides as emerging therapeutics for lipid disorders

Bioactive peptides derived from endogenous proteins, engineered mimetics, and food hydrolysates exert diverse actions on lipid metabolism. Short amphipathic helices modeled on apolipoprotein A-I have been shown to promote cholesterol efflux, reduce inflammation, and limit atherogenesis in multiple preclinical studies (Xie et al., 2010; Morgantini et al., 2010). HDL mimetics and apoA-I containing particles progressed into human studies (clinical evidence), illustrating translational intent though outcomes were mixed (Kootte et al., 2015; Nicholls et al., 2018). Parallel approaches seek to inhibit proatherogenic proteins such as PCSK9 using cyclic or macrocyclic peptides that block the PCSK9–LDL receptor interaction, and some of these leads have demonstrated oral bioavailability or potent *in vivo* activity after optimization (clinical evidence) (Johns et al., 2023; Burnett and Hooper, 2023). In addition, an expanding literature documents naturally derived peptides from plants, legumes, dairy, seafood, and fermented foods that reduce cholesterol absorption, modulate bile acid synthesis, or upregulate LDL receptor pathways in cellular and animal models (preclinical evidence) (Wang et al., 2023; Lee et al., 2021; Lammi et al., 2014). These diverse mechanisms offer opportunities to complement existing lipid lowering strategies.

### 1.3 Limitations of experimental peptide discovery

Conventional discovery pipelines rely on fractionation, biochemical assays, animal models, and iterative medicinal chemistry. Such workflows are resource intensive and frequently yield leads that fail because of poor stability, low oral bioavailability, rapid clearance, or lack of activity in humans. Clinical experience underscores this translational gap. For example, early apoA-I mimetics produced robust preclinical benefit but did not uniformly improve HDL functional biomarkers in small human trials (Watson et al., 2011; Navab et al., 2005). HDL mimetic infusions showed plaque targeting in some investigations but delivered variable clinical signal across studies (Zheng et al., 2016; Hovingh et al., 2015). Moreover, natural peptides often require sequence optimization to resist proteolysis and to achieve sufficient plasma exposure, a constraint that raises development costs (Sherman et al., 2010; Zhang et al., 2022).

### 1.4 Role of computational approaches in peptide discovery and function prediction

Computational methods now occupy a central role in accelerating peptide lead discovery and in prioritizing sequences for experimental validation. At the sequence level, machine learning classifiers and deep learning frameworks can predict peptide bioactivity and peptide-protein interactions with increasing accuracy, enabling high throughput *in silico* screening of candidate libraries (Xie et al., 2025). Early tools such as PeptideLocator and more recent deep learning frameworks for peptide–protein interaction prediction illustrate this trajectory (Mooney et al., 2013; Lei et al., 2021). Ensemble and generative models have been applied to generate novel peptide sequences with desired functional signatures and to learn sequence motifs associated with activity (Zhang et al., 2024; Wan et al., 2022). Structure informed approaches using molecular docking and molecular dynamics provide mechanistic hypotheses for peptide binding to enzymatic targets or membrane interfaces, and coupling these methods with *in vitro* assays has already identified cholesterol esterase inhibitors and other lipid-modulating peptides from food sources (Wang et al., 2023; Wang P. et al., 2024).

Computational optimization addresses key liabilities of therapeutic peptides. Rational design guided by molecular modeling and mutational scans has improved affinity and serum stability for PCSK9 inhibitory peptides and for apoA-I mimetics. Conjugation strategies informed by modeling, such as albumin-binding fusions or lipid tags, increase half life and *in vivo* potency (Zhang et al., 2022; Nguyen et al., 2015). Macrocyclization and constrained scaffolds that emerged from *in silico* screening have produced orally bioavailable leads that block protein–protein interactions relevant to lipid homeostasis (Johns et al., 2023; Burnett and Hooper, 2023).

Complementary computational pipelines integrate proteomics, peptidomics, and mass spectrometry with machine learning to reveal bioactive fragments directly from complex biological samples. These hybrid workflows accelerate candidate prioritization by combining predictive scores with experimental evidence of natural occurrence and cleavage compatibility (Madsen et al., 2022; Giguère et al., 2015). The same principles apply in nutrition science where AI assisted screening identifies food protein regions likely to yield cholesterol lowering peptides after gastrointestinal digestion. Subsequent docking and cell assays confirm mechanistic effects on cholesterol micellization, bile acid pathways, or hepatic lipid handling (Bao et al., 2022; Yuan et al., 2025).

Taken together, the literature shows that computational discovery and functional prediction substantially compress the search space for lipid-active peptides and provide tractable strategies to overcome proteolytic instability and delivery barriers. Continued progress will require richer training data sets drawn from high quality experimental studies, standardized activity annotations, and iterative validation cycles that bridge *in silico* predictions and rigorous *in vitro* and *in vivo* testing. Recent studies that combine virtual screening, molecular dynamics, and experimental validation serve as practical templates for such integrative pipelines (Zhu et al., 2025; Wang Y. et al., 2024).

## 2 Biological roles of bioactive peptides in lipid metabolism and cardiovascular diseases

### 2.1 Endogenous peptides involved in lipid homeostasis

**FIGURE 1:**
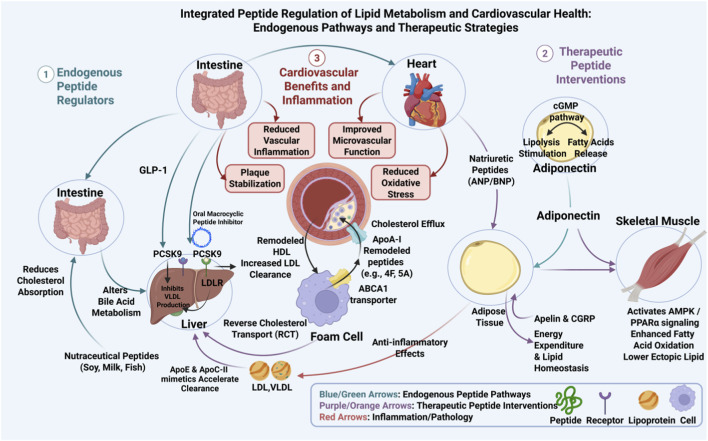
Integrated regulatory pathways of endogenous and therapeutic peptides in lipid metabolismand cardiovascular protection. Blue/greenrepresentendogenouspeptide-mediated regulatory pathways; arrowspurple/orange arrows indicate therapeutic peptide intervention strategies, red arrows denoteinflammatory and pathological processes. Key peptides and their targets, as well as coremolecular events (e.g., cholesterol efflux, reverse cholesterol transport, lipid oxidation) inlipid homeostasis and anti-atherosclerosis, are depicted across major metabolic tissues (intestine, adipose tissue, liver, skeletal muscle, heart).

A diverse set of endogenous peptides operate as hormonal and paracrine regulators of lipid flux and lipoprotein metabolism (Figure 1). Glucagon like peptide 1 influences intestinal chylomicron output and postprandial triglyceride excursions, and GLP 1 receptor agonists reduce postprandial lipemia and modulate hepatic very low density lipoprotein production in experimental and clinical studies (Farr et al., 2014; Matikainen et al., 2019). Adiponectin, secreted from adipose tissue, activates AMP activated protein kinase and PPAR alpha signaling in liver and muscle to enhance fatty acid oxidation and to lower ectopic lipid accumulation; these signaling events link adiponectin to favorable lipid profiles and reduced cardiometabolic risk (Yanai and Yoshida, 2019; Yoon et al., 2006). Cardiac natriuretic peptides stimulate adipocyte lipolysis through a cGMP dependent pathway that complements classical adrenergic control, thereby contributing to systemic lipid mobilization and postprandial lipid oxidation (Sengenès et al., 2000; Wang, 2012). Peptides such as apelin and calcitonin gene related peptide also influence adipocyte biology, fatty acid handling, and energy expenditure, and growing experimental literature ties these mediators to lipid homeostasis and cardiac metabolism (Bertrand et al., 2015; Liu et al., 2017).

**FIGURE 2:**
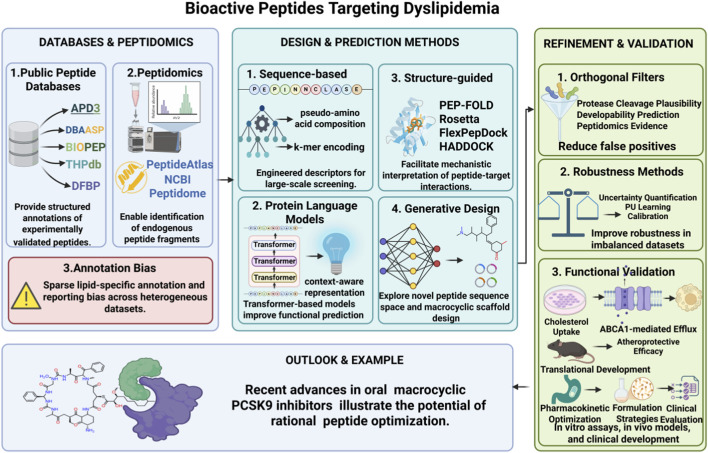
Schematic workflow of computational discovery, refinement, and translational application ofbioactive peptides targeting dyslipidemia. Key components include peptide databases and peptidomics resources, core computationaldesign/prediction methods, robustness improvement strategies, functional validation, andtranslational development, with orally bioavailable macrocyclic PCSK9 inhibitors as arepresentative successful example.

### 2.2 Therapeutic peptides targeting dyslipidemia

Therapeutic strategies exploit both mimics of endogenous apolipoproteins and distinct peptide scaffolds that block pathogenic protein interactions. Apolipoprotein A I mimetic peptides, including 4F and 5A series, enhance cholesterol efflux via ABCA1 and remodel HDL to improve reverse cholesterol transport in animal models; several studies document reduced atherosclerotic burden after peptide administration (preclinical evidence) (Xie et al., 2010; Amar et al., 2010). Clinical translation of HDL mimetics has produced mixed results; infusion studies with CER 001 revealed biological effects on plaque lipid content in some cohorts while larger trials yielded neutral outcomes, indicating challenges in delivery and patient selection (clinical evidence) (Tardy et al., 2014; Tardif et al., 2014). ApoE and apoC II derived mimetic peptides accelerate clearance of atherogenic lipoproteins and normalize triglyceride handling in dyslipidemic models, offering complementary routes to lower atherogenic particle burden (preclinical evidence) (Komatsu et al., 2019; Anantharamaiah et al., 2018).

Recently developed macrocyclic and constrained peptides targeting PCSK9 represent a different therapeutic paradigm. Oral macrocyclic inhibitors that block PCSK9 binding to the LDL receptor achieved potent LDL lowering in early clinical studies, demonstrating that peptide scaffolds can be optimized for oral exposure and high affinity against protein protein interfaces (clinical evidence) (Johns et al., 2023; Ballantyne et al., 2023). In parallel, nutraceutical research identifies food derived peptides from soy, milk casein, fish and other sources that reduce cholesterol absorption, alter bile acid metabolism, or modulate hepatic lipid regulatory genes *in vitro* and *in vivo*, suggesting routes for dietary peptide intervention in dyslipidemia (preclinical evidence) (Lee et al., 2021; Jiang et al., 2020).

### 2.3 Mechanisms of action in cardiovascular protection

Bioactive peptides confer cardiovascular benefit through mechanisms that extend beyond simple lipid lowering. ApoA I mimetics promote macrophage cholesterol efflux via ABCA1 mediated pathways and can augment LCAT activity, thereby accelerating reverse cholesterol transport and reducing lipid retention within plaques. These molecular actions are accompanied by reductions in lipoprotein oxidation and restoration of HDL anti inflammatory capacity in animal models (preclinical evidence) (Amar et al., 2010; Chen et al., 2009). Peptides that target PCSK9 increase LDL receptor recycling and peripheral LDL clearance, producing robust decreases in circulating LDL cholesterol that translate to plaque and event risk reduction in clinical paradigms when applied with established therapies (clinical evidence) (Ballantyne et al., 2023; Landmesser and Makhmudova, 2023).

Peptide modulators also act on intestinal and hepatic axes of cholesterol handling. Casein and soy derived sequences upregulate trans intestinal cholesterol excretion and alter bile acid synthesis through effects on intestinal transporters and fibroblast growth factor signaling, which increases fecal sterol loss and lowers systemic cholesterol in murine models (preclinical evidence) (Lee et al., 2021; Lee and Youn, 2020). Anti inflammatory and endothelial effects are another unifying theme. GLP 1 receptor agonists reduce adipose inflammation and improve microvascular function while apolipoprotein mimetics attenuate vascular inflammation and oxidative stress, thereby stabilizing plaques independent of changes in plasma lipid concentrations (clinical evidence) (Hjerpsted et al., 2018). Adiponectin further protects vascular cells by enhancing nitric oxide bioavailability and suppressing smooth muscle proliferation, linking metabolic peptide signaling to preserved vascular integrity (Yanai and Yoshida, 2019).

Collectively, experimental evidence supports a model in which peptide interventions alter lipoprotein composition, accelerate cholesterol removal, suppress vascular inflammation and favorably remodel metabolic tissues. Translational success will depend on overcoming delivery and stability constraints, selecting appropriate patient populations, and combining computational lead optimization with rigorous *in vitro* and *in vivo* validation. Representative primary studies and trials cited above provide a rich evidence base for these mechanistic and translational claims (Navab et al., 2005; Ballantyne et al., 2023; Lee and Youn, 2020; Navab et al., 2005).

## 3 Data resources for computational peptide discovery

### 3.1 Peptide sequence and function databases

Public databases form the foundation for *in silico* peptide discovery because they supply the primary sequences, annotated activities, experimental conditions, and, in some cases, structural models that machine learning and physics-based methods consume. Dedicated antimicrobial peptide repositories remain the most mature and richly annotated category (Table 1). Well established resources collect thousands of experimentally validated sequences with activity readouts and metadata; examples include the Antimicrobial Peptide Database update APD3, the Database of Antimicrobial Activity and Structure of Peptides DBAASP, the CAMP family of AMP resources, and the DRAMP collection (Wang et al., 2016; Pirtskhalava et al., 2021; Waghu et al., 2014). These resources provide curated sequences, minimal inhibitory concentrations, chemical modifications and structural entries that are commonly re-used as positive training data in predictive models.

**TABLE 1:** Public databases supporting computational discovery of bioactive peptides.

Category	Database	Main focus	Core content	Curation level	Label type	Assay standardization	Cardiovascular relevance	Key bias/limitation
Antimicrobial peptide databases	APD3	Curated antimicrobial peptides	Experimentally validated sequences, activity data (e.g., MIC), physicochemical and structural annotations	Manual curation (literature-based)	Quantitative + qualitative	Low–moderate (heterogeneous MIC assays)	Low (primarily antimicrobial)	Strong AMP bias; limited lipid-related annotations; scarce true negatives
	DBAASP	Antimicrobial activity and structure	Quantitative activity values, assay conditions, structural data, peptide modifications	Manual + semi-automated curation	Quantitative (MIC, IC50)	Moderate (assay metadata recorded but not standardized)	Low	Bias toward antimicrobial assays; condition variability affects comparability
	CAMP	AMP collection with prediction tools	Natural/synthetic AMP sequences, classification tools, structural links	Mixed (manual + automated integration)	Mostly qualitative	Low	Low	Includes predicted entries → label noise; limited experimental validation
	DRAMP	Comprehensive AMP repository	Natural, synthetic, patented, clinical AMPs with curated annotations	Manual curation (extensive)	Qualitative + partial quantitative	Low–moderate	Low	Overrepresentation of antimicrobial peptides; sparse negative data
Therapeutic peptide databases	THPdb	Approved and investigational therapeutic peptides	FDA-approved peptides, drug variants, indications, pharmacological data	Manual expert curation	Qualitative (clinical annotation)	High (clinical-grade data)	Moderate–high (includes metabolic/cardiovascular drugs)	Limited size; lacks mechanistic granularity and negative outcomes
Food- and nutraceutical-derived peptide databases	DFBP	Food-derived bioactive peptides	Peptides from dietary proteins with reported bioactivities	Manual (literature extraction)	Mostly qualitative	Low	Moderate (lipid metabolism-related entries exist)	Predominantly preclinical data; heterogeneous assays; publication bias
	FermFooDb	Fermented food peptides	Peptides from fermented foods with functional annotations	Manual + database integration	Qualitative	Low	Moderate	Limited mechanistic validation; mostly *in vitro* evidence
Proteomics and peptidomics repositories	PeptideAtlas	MS-validated peptide identifications	High-confidence peptide-spectrum matches mapped to proteomes	Automated pipeline + expert validation	Structural/presence evidence	High (MS standards)	Indirect	No functional labels; requires downstream inference
	PRIDE/ProteomeXchange	Public proteomics datasets	Raw and processed MS data with metadata	Community submission + QC pipelines	Structural/experimental metadata	High (standardized formats, variable experiment design)	Indirect	Highly heterogeneous datasets; annotation inconsistency
Protease and cleavage databases	MEROPS	Proteases and substrate specificity	Protease classification, cleavage patterns, substrates and inhibitors	Manual expert curation	Structural + functional relationships	Moderate	Indirect	Focus on proteolysis, not peptide function; limited activity labels

Therapeutic peptide repositories complement AMP databases by focusing on clinically relevant sequences and approved peptide drugs. THPdb catalogs FDA approved peptide and protein therapeutics and their variants, while several recent efforts aim to compile large, multi-function collections of experimentally validated therapeutic peptides suitable for machine learning (Usmani et al., 2017). Food-derived and nutraceutical peptide databases such as DFBP and FermFooDb assemble peptides discovered from dietary proteins and fermented foods together with reported bioactivities, including some entries annotated for lipid-modulating effects (Qin et al., 2022). These specialized resources enable cross-domain mining for sequences with potential cardiometabolic utility.

Proteomics and peptidomics repositories offer orthogonal evidence of natural peptide occurrence. PeptideAtlas and the NCBI Peptidome archive mass spectrometry identifications mapped to proteomes, which allows researchers to link candidate bioactive fragments to endogenous cleavage events and tissue contexts (Desiere et al., 2006; Ji et al., 2010). Databases that describe proteases and cleavage specificity, such as MEROPS, assist in modeling how precursor proteins can yield short bioactive peptides *in vivo* (Rawlings et al., 2002). Domain specific peptide collections, for example, peptidome resources for particular diseases, demonstrate how experimental peptidomics can seed discovery pipelines (Krochmal et al., 2016).

Public peptide databases differ substantially in curation depth, label quality, and downstream utility. AMP-centric repositories such as APD3, DBAASP, CAMP, and DRAMP are rich in experimentally validated sequences and quantitative activity annotations, but they are not optimized for lipid-modulating peptides. THPdb focuses on approved and investigational therapeutics, whereas PeptideAtlas, PRIDE, and MEROPS provide orthogonal evidence of peptide occurrence or proteolytic plausibility rather than functional labels. As a result, models trained on these resources may appear strong on internal benchmarks yet still fail to generalize to cardiovascular peptide tasks because the label space, assay conditions, and sequence distributions are all shifted. This limitation is particularly important for lipid-active peptides, where many positive examples come from small studies with heterogeneous endpoints and very limited negative or neutral reporting.

### 3.2 Cardiovascular- and lipid-related peptide datasets

Compared to antimicrobial or general therapeutic categories, data sets explicitly annotated for lipid modulation and cardiovascular action are sparse and heterogeneous. Food-derived peptide studies report numerous sequences that alter cholesterol absorption, inhibit HMG-CoA reductase, or modulate intestinal transporters, yet these reports are dispersed across small experiments and diverse model systems (Qin et al., 2022; Karami and Akbari-Adergani, 2019). Aggregated databases of food bioactive peptides include cholesterol-related annotations, but the entries are fewer and often lack standardized activity metrics, which complicates direct comparisons and model training. The literature contains many individual primary studies that identify cholesterol-lowering dipeptides and short motifs from legumes, dairy, fish and plant seeds, but a centralized, high quality compendium of lipid-active peptides remains limited.

Apolipoprotein mimetic peptides binding scaffolds supply another class of lipid-relevant sequences. These designs are well documented in primary research articles that report sequence variants, structure activity relationships and *in vivo* lipid endpoints (Mendez et al., 1994; Zhang et al., 2020). However, these data are usually published as discrete experimental reports rather than integrated machine-readable data sets, which reduces their immediate utility for data hungry algorithms. Peptidomics studies of plasma, HDL particles and atherosclerotic tissue sometimes reveal endogenous fragments with putative cardiovascular roles, but sample sizes and annotation completeness are variable across studies.

### 3.3 Challenges in data quality and annotation

Several interrelated data problems undermine robust computational discovery. First, functional annotation bias is pervasive across biological databases. A minority of experimental studies produce many annotations, which concentrates evidence on a small set of proteins or peptides and skews downstream learning. This “few studies, many annotations” phenomenon affects peptide resources as well and promotes overfitting of models to well studied sequence families (Schnoes et al., 2013).

Second, construction of negative samples for supervised learning carries major pitfalls. Many AMP and general peptide predictors use negative sets sampled from UniProt sequences that lack any annotated activity. Such negatives may unintentionally include uncharacterized bioactive peptides or differ systematically in length and composition from positives (Sidorczuk et al., 2022). Recent work shows that benchmarking and model performance are highly sensitive to negative sampling strategies, and that biased negative sets produce inflated accuracy estimates (Li et al., 2025). Careful negative set design, or use of positive-unlabeled learning paradigms, is therefore essential.

Third, the biological properties of peptides intensify prediction difficulty. Short peptide length combined with high sequence variability yields a weak signal to noise ratio for motif extraction. A ten amino acid fragment may tolerate many substitutions while retaining function, and small changes can also abolish activity. Models that rely purely on sequence statistics struggle with this combinatorial variability. Peptidomics measurement variability and incomplete metadata further complicate mapping sequence to function (Baggerman et al., 2004; Coelho et al., 2024).

Fourth, lipid-related peptide data are particularly scarce relative to other functional categories. Although food science and individual mechanistic studies report cholesterol-modulating peptides, these records are often small, lack uniform activity units and fail to report negative or neutral findings (Zhao et al., 2025). This sparsity constrains supervised learning and increases the risk of overoptimistic conclusions from limited samples. Integrative strategies that combine curated literature curation, peptidomics evidence and physics-aware modeling can partially mitigate data scarcity by adding orthogonal constraints to purely data driven approaches (Nordquist et al., 2023).

In sum, the current ecosystem offers valuable repositories and many primary data points, but it is also marked by annotation bias, problematic negative sampling, and limited lipid-specific collections. Addressing these issues is a prerequisite for reliable peptide activity prediction, and it motivates hybrid pipelines that merge curated databases, careful negative sampling, experimental peptidomics and mechanistic modeling.

## 4 Computational methods for bioactive peptide discovery

### 4.1 Sequence-based prediction models

Sequence-based approaches remain the first line of computational triage owing to their speed and scalability, enabling screening of millions of candidate fragments derived from proteomes or enzymatic digests. Early scoring schemes such as PeptideRanker were designed to prioritize potentially bioactive peptides (Mooney et al., 2012); many successful classifiers employ engineered descriptors (e.g., pseudo amino acid composition, physicochemical indices, k-mer frequency profiles) combined with traditional machine learning algorithms like support vector machines and random forests—a strategy formalized for antimicrobial peptides in tools such as iAMP-2L and iAMPpred (Xiao et al., 2013; Meher et al., 2017)—while alignment-free quantitative sequence–activity models enhance robustness when homologous templates are scarce and reduce dependence on multiple sequence alignments that poorly capture short, variable peptides (Pinacho-Castellanos et al., 2021). Recent progress replaces handcrafted features with representation learning from large protein language models, where BERT-style encodings and hybrid CNN–BiLSTM architectures achieve higher sensitivity in discriminating small bioactive peptides from background sequences (Xing et al., 2023; Almotairi et al., 2024); another key direction predicts the proteolytic release of candidate peptides from precursors to focus on fragments likely to exist *in vivo* and thus reduce false positives, as exemplified by DeepPeptide for cleavage-site prediction (Teufel et al., 2023). The databases that underpin these sequence models include comprehensive AMP collections and food-derived peptide catalogs supplying labeled positives, with APD3, DBAASP, BIOPEP and THPdb widely used for training and benchmarking (Wang et al., 2016; Pirtskhalava et al., 2021; Usmani et al., 2017; Minkiewicz et al., 2019).

Despite these advances, reported performance of sequence-based models should be interpreted cautiously because peptide prediction benchmarks are highly sensitive to the construction of negative datasets. Many studies rely on randomly sampled or artificially generated negatives, which can inflate apparent accuracy and even alter model ranking across methods. As a result, models evaluated under different negative sampling strategies are often not directly comparable. Future benchmarking should therefore report multiple metrics, including AUROC, AUPRC, and Matthews correlation coefficient (MCC), and prioritize evaluation on external or experimentally validated datasets under matched sampling protocols rather than relying solely on internal cross-validation (Sidorczuk et al., 2022).

### 4.2 Structure-based and structure-aware approaches

Structure-aware methods address the intrinsic challenge that many peptides fold or adopt ordered conformations only upon binding, necessitating explicit conformational sampling for induced-fit modelling—exemplified by Rosetta FlexPepDock’s *ab initio* folding and docking protocol (Raveh et al., 2011). To improve success rates on flexible peptides, tools coupling ensemble generation with information-driven docking (e.g., HADDOCK pipelines) expand the conformational search space prior to refinement (Geng et al., 2017; Charitou et al., 2022), while rapid tertiary structure predictors like PEP-FOLD3 supply practical starting conformations for downstream docking or molecular dynamics (Lamiable et al., 2016). The advent of high-accuracy protein structure predictors such as AlphaFold2 has shifted the landscape by delivering high-quality receptor models and, when combined with fragment modelling, reasonable peptide backbone placements that frequently serve as inputs to docking or network-based scoring (Jumper et al., 2021). Post-docking scoring using physics-informed estimators (MM-PBSA, dMM-PBSA) re-ranks candidate poses and improves correlation with experimental affinities (Spiliotopoulos et al., 2016), while molecular dynamics simulations provide atomistic insight into peptide interactions with membranes and lipoprotein surfaces—particularly relevant for cholesterol-modulating peptides acting at lipid interfaces (Heller, 2024; Zhan and Lazaridis, 2012). Specialized binding-site predictors such as PepSite, PEP-SiteFinder and PepBind further localize plausible peptide-binding regions on protein surfaces to guide targeted docking runs (Trabuco et al., 2012; Saladin et al., 2014; Das et al., 2013).

### 4.3 Machine learning and deep learning models

Deep learning architectures have rapidly overtaken classical pipelines for peptide property prediction by learning hierarchical features directly from raw sequences or structures. Attention-based models such as PepNN and transformer contrastive frameworks like PepBCL predict peptide binding sites and binding propensity from sequence and structure inputs, demonstrating the power of self-attention mechanisms (Abdin et al., 2022; Wang R. et al., 2022). Convolutional neural networks, which consume encoded sequence maps or residue pair matrices, effectively capture local interaction motifs, while transformers model long-range dependencies and context within variable-length peptides, addressing the inherent variability of peptide sequences (Almotairi et al., 2024; Chandra et al., 2023). Graph neural networks operating on residue contact graphs naturally represent three-dimensional neighborhood relationships and have shown competitive performance in peptide-protein interface prediction, as evidenced by recent comparative studies (Zhou et al., 2022; Pancino et al., 2024). Hybrid strategies that combine structure and sequence encoders, leverage transfer learning from large protein language models, and apply contrastive or multi-task losses have improved generalization on small peptide datasets, addressing data scarcity (Yin et al., 2024; Zhu et al., 2024). Explainability and model calibration are active research fronts, as confidence and interpretability are crucial for guiding experimental follow-up; ensemble and uncertainty-aware frameworks reduce spuriously confident predictions when training data are sparse, enhancing overall reliability (Wang G. et al., 2022).

From a comparative perspective, sequence-based models offer high throughput and scalability but limited mechanistic interpretability, whereas structure-based and hybrid models provide greater target-level insight at higher computational cost. Deep learning approaches can improve predictive accuracy, particularly for complex sequence–structure relationships; however, their performance remains strongly dependent on training data quality, label balance, and dataset size. In small and biased peptide datasets, even advanced architectures may fail to generalize beyond benchmark settings, underscoring the need for careful dataset design and evaluation.

### 4.4 Integrative omics-based discovery strategies

Integrative pipelines combine peptidomics, proteomics, transcriptomics and computational scoring to prioritize candidates that are both predicted active and experimentally observed in biological samples. Public resources such as PeptideAtlas supply orthogonal evidence of peptide occurrence to filter artefactual *in silico* hits (Deutsch, 2010). Protease databases like MEROPS further restrict candidates through *in silico* digestion simulations that model plausible *in vivo* release pathways (Rawlings et al., 2008). Workflow case studies confirm that combining peptidomics evidence, cleavage prediction, sequence activity models and docking rescoring achieves higher validation rates than any single method alone (Teufel et al., 2023; Raveh et al., 2011). Such integrative strategies are especially critical for domains with sparse labeled data, such as lipid-modulating peptides, where triangulating evidence from orthogonal data types mitigates label scarcity and reduces false discovery (Pirtskhalava et al., 2021). The major computational paradigms discussed above are summarized in Table 2. Importantly, integrative strategies partially mitigate biases introduced at the sequence-model level, including those arising from negative sampling and dataset imbalance. By incorporating orthogonal evidence such as peptide detectability, proteolytic plausibility, and structural compatibility, these workflows reduce reliance on purely statistical discrimination and improve the robustness of candidate prioritization. Nevertheless, the overall predictive reliability still depends on the consistency and quality of the underlying datasets, and systematic benchmarking across integrated pipelines remains limited.

**TABLE 2:** Overview of computational strategies for bioactive peptide discovery.

Strategy	Core focus	Strengths	Limitations
Sequence-based models	Predict activity from primary sequence features	Fast, scalable, suitable for large-scale screening	Limited structural insight; prone to false positives
Structure-aware approaches	Model peptide–target interactions	Mechanistic interpretability; improved binding prediction	Computationally intensive; peptide flexibility challenges
Machine and deep learning	Learn complex sequence–structure patterns	High predictive performance; adaptable to diverse tasks	Data dependency; interpretability concerns
Integrative omics strategies	Combine prediction with experimental evidence	Higher biological relevance; reduced false discovery	Workflow complexity; multi-source data requirement

## 5 Functional prediction of peptides targeting dyslipidemia and cardiovascular diseases

For cardiovascular peptide discovery, the central question is not only how to predict activity, but how to prioritize candidates most likely to affect lipid metabolism and atherosclerosis. Sequence-based models are useful for high-throughput triage, structure-based rescoring provides greater target-level insight for mechanistic interpretation, and multi-label or multi-task learning is particularly valuable when lipid-lowering, anti-inflammatory, and anti-foam-cell activities co-occur in the same peptide. In this setting, sequence-only, structure-aware, and deep-learning approaches should be compared explicitly in terms of scalability, interpretability, and dependence on training data quality.

Mechanistic targets validated in cell and animal studies—such as modulation of intestinal ABCA1, HMG-CoA reductase, or macrophage cholesterol efflux—provide experimentally grounded endpoints for *in silico* screening (Banno et al., 2019; Lin et al., 2015). Anti-atherosclerotic activity often arises from combined effects on lipid flux, foam cell formation, oxidative stress, and inflammatory signaling. Peptides that mimic pro-resolving mediators or reduce macrophage lipid uptake serve as functional labels for model training (Tang et al., 2024; Santos-Sánchez et al., 2022a). Multi-label and multi-task frameworks, including hierarchical systems like AMAP and MultiPep, enable simultaneous prediction of overlapping biological activities, improving real-world prioritization for cardiovascular applications (Gull et al., 2019; Grønning et al., 2021). Advanced strategies using contrastive learning, meta-learning, and task-aware loss functions further enhance generalization to underrepresented functional classes, addressing the scarcity of rare lipid-modulating peptide data (Zhou et al., 2025).

Practical pipelines should integrate sequence-level scoring, structure-based rescoring against validated targets (e.g., PCSK9, NPC1L1, CETP, scavenger receptors), peptidomics evidence to confirm *in vivo* occurrence, and functional classifiers for lipid-lowering, anti-inflammatory, or anti-foam-cell activity. These combined approaches produce higher hit rates than any single method alone and allow iterative prioritization of peptides with the greatest translational potential (Johns et al., 2023; Cho et al., 1998).

## 6 Experimental validation and translational considerations

### 6.1 From in silico prediction to *in vitro* and *in vivo* validation

Computational models that predict bioactive peptide candidates must be rigorously validated with empirical evidence because *in silico* scores alone cannot assure biological effectiveness in complex physiological systems. A common first step is *in vitro* testing using biochemical assays to evaluate peptide effects on cholesterol homeostasis, lipoprotein interactions, or receptor binding. For example, peptides designed to mimic apolipoprotein A-I (apoA-I) have been shown to enhance cholesterol efflux from macrophages and reduce oxidative stress in cultured vascular cells, which supports their predicted roles in atheroprotection (Gou et al., 2020).

Cellular assays using hepatocytes or enterocytes can assay interaction with key regulators such as PCSK9 or lipid uptake transporters, providing mechanistic context for peptide functionality. Validated PCSK9 inhibitory peptides demonstrate strong affinity and significant inhibition of the PCSK9–LDL receptor interaction in biochemical and cellular binding assays (Tombling et al., 2021).

*In vivo* validation is the next essential layer. Hypolipidemic activity predicted *in silico* must translate into measurable modulation of systemic lipid levels, plaque burden, or inflammatory indices in animal models. Novel apoA-I mimetic peptides were demonstrated to reduce atherosclerotic lesion formation and promote physiological HDL function in ApoE^−/−^ mice, consistent with enhanced reverse cholesterol transport (Gou et al., 2020).

Vaccine strategies based on PCSK9 peptides have been evaluated in multiple rodent models and have induced robust, long-lasting antibody responses with corresponding reductions in total cholesterol and LDL-C levels. In preclinical studies of peptide-based PCSK9 vaccines, immunized mice exhibited LDL-C lowering up to 50%, and the cholesterol lowering persisted for more than 1 year, validating the functional predictions of these designed immunogens (Galabova et al., 2014).

Besides lipid lowering, *in vivo* models also test effects on inflammation and vascular biology. Peptides that modulate immune responses and lipid metabolism can be validated through flow cytometry and histological analyses showing reduced inflammatory cell infiltrates, increased regulatory T cell fractions, and decreased expression of pro-inflammatory genes in atherosclerotic lesions (Tang et al., 2024).

Natural and food-derived peptides predicted to have lipid modulation properties have also been validated *in vivo*. Lupin protein hydrolysate peptides significantly reduced plasma lipid levels, lowered cardiovascular and atherogenic risk indices, and alleviated endothelial permeability changes in Western diet–fed ApoE^−/−^ mice, demonstrating how computationally derived activities can forecast physiological outcomes (Santos-Sánchez et al., 2022a).

### 6.2 Preclinical evidence in dyslipidemia and atherosclerosis models

Robust preclinical evidence supports efficacy of several classes of peptide therapeutics for dyslipidemia and related cardiovascular disorders. Apolipoprotein mimetic peptides have a long history of validation in animal models. Episodic administration of designed apoA-I mimetic peptides reduced atherosclerotic lesion formation and improved functional measures of HDL in transgenic mice deficient in apolipoproteins or on high-cholesterol diets, aligning with predictions from computational design of amphipathic helical sequences (Getz and Reardon, 2011).

Variants of apoE-based peptides have reversed hyperlipidemia and reduced plaque burden in LDL receptor null and ApoE null mice, illustrating that mimetics of endogenous regulators can produce broad effects on lipoprotein metabolism and inflammation (Osei-Hwedieh et al., 2011).

Emerging research also demonstrates that peptide vaccines targeting PCSK9 can ameliorate dyslipidemia in rodent models. Virus-like particle (VLP) based PCSK9 peptide vaccines elicited high titer anti-PCSK9 IgG antibodies, decreased total cholesterol, free cholesterol, and triglycerides in mice, and showed consistent effects across different PCSK9 epitopes. These vaccines reduced lipid levels more than 40% in several models, confirming functional predictions of immune-based lipid modulation (Crossey et al., 2015).

Hydrolysates containing multiple peptides also provide preclinical evidence. In hypercholesterolemic mice, lupin protein hydrolysate reduced hepatic cholesterol and triglyceride levels by modulating key enzymes in the LDL receptor and PCSK9 pathways (Santos-Sánchez et al., 2022b). Beyond lipid levels, peptide interventions impact atherosclerotic inflammation and tissue pathology. Experimental peptide-based therapies have reduced foam cell formation, modified macrophage phenotypes, and lowered endothelial dysfunction in rodent models, which aligns with mechanisms predicted by multi-target computational screens that combine lipid modulation with anti-inflammatory actions (White et al., 2022).

### 6.3 Challenges in clinical translation

Despite promising preclinical data, clinical translation of peptide-based therapies for dyslipidemia and atherosclerosis faces several hurdles. Pharmacokinetic limitations remain a significant barrier because short peptides are often rapidly degraded *in vivo*, requiring chemical stabilization strategies to achieve therapeutic exposure. Article reviews highlight that many apoA-I mimetic peptides with *in vivo* efficacy still have poor half-life and require formulation advances to become clinically viable (Wolska et al., 2021). Moreover, achieving consistent and sustained target engagement is challenging. For peptide vaccines against PCSK9, the longevity of antibody responses varies with vaccine constructs and adjuvants, and initial declines in antibody levels may necessitate periodic boosting to maintain LDL-C lowering effects—a complexity not yet solved in current formulations (Chackerian and Remaley, 2024). The safety profile of peptide modalities also demands rigorous evaluation. Although toxicity assessments in rodents for some anti-PCSK9 peptide vaccines have shown no significant adverse effects across major organ systems, long-term safety and immunogenicity must be evaluated in humans to anticipate rare immune responses and off-target effects (Momtazi-Borojeni et al., 2023).

Another translational challenge is heterogeneity in human dyslipidemia and cardiovascular disease. Genetic variability in lipoprotein metabolism and immune response means that peptides effective in homogeneous animal models may not translate directly to diverse human populations that vary in PCSK9 expression, LDL receptor polymorphisms, and inflammatory profiles. Human clinical studies of HDL targeted therapies have sometimes failed to replicate animal model benefits despite promising mechanistic rationale (Uehara et al., 2015). Clinical translation is best illustrated by contrastive examples. The oral macrocyclic PCSK9 inhibitor MK-0616 achieved robust LDL-C lowering in a phase 2b study, with placebo-adjusted reductions of up to 60.9% at week 8, demonstrating that peptide scaffolds can be engineered for oral exposure, high affinity, and strong target engagement (clinical success) (Ballantyne et al., 2023; Zheng et al., 2020). By contrast, CER-001, an HDL mimetic peptide, showed promising plaque-targeting or proof-of-concept signals in early open-label studies, but subsequent randomized trials failed to demonstrate a significant effect on carotid wall thickness, highlighting the gap between mechanistic plausibility and clinical efficacy (clinical failure) (Kootte et al., 2015; Johns et al., 2023). These examples underscore that favorable preclinical pharmacology does not guarantee human efficacy, and that careful selection of patient populations, dosing strategies, and mechanistic readouts is essential for translational success.

Regulatory pathways for peptide therapeutics can be complex because they straddle biologic and small molecule frameworks, especially when modified residues or noncanonical structures are involved. Peptides with nonstandard amino acids or macrocyclic scaffolds may require additional biopharmaceutical characterization to satisfy regulatory authorities. Integration of rigorous ADME (absorption, distribution, metabolism, excretion) and immunogenicity assessment early in development is therefore essential for translational success.

Finally, cost and manufacturability remain practical barriers. Large peptides and peptide vaccines often involve complex synthesis or display technologies, which can raise production cost. Balancing therapeutic potency, stability, and manufacturability is a key translational consideration and demands close collaboration between computational designers and process chemists.

## 7 Future perspectives

### 7.1 Data infrastructure and standardized peptide repositories

Future work should prioritize lipid-focused, assay-standardized peptide repositories with explicit annotation of peptide source, experimental system, dose, endpoint, and negative or neutral outcomes. As discussed in Section 3.3, standardized annotation is essential for reducing dataset fragmentation and improving model transferability. Balanced coverage across functional classes and transparent reporting of database versions and filtering thresholds will further enhance the utility of peptide resources for machine learning applications.

### 7.2 Advances in machine learning models for peptide discovery

Advanced modeling techniques, including pretrained protein language models, meta-learning, few-shot learning, and contrastive approaches, will continue to improve generalization across short, variable peptides with low-information content (Rahman et al., 2016; Wu et al., 2023). Multi-label and multi-task frameworks capture co-occurring lipid-lowering, anti-inflammatory, and antioxidant activities, while task-aware loss functions and semantic-preserving perturbations mitigate data imbalance and overfitting (Fang et al., 2025). Future pipelines should explicitly quantify uncertainty and evaluate model generalizability using domain-specific held-out sets and external peptidome evidence.

### 7.3 Integration of computational and experimental workflows

To reduce false positives and accelerate discovery, future workflows will integrate multiple orthogonal filters prior to synthesis, including peptidomics occurrence, lipoprotein proteomes, protease cleavage prediction, structure-aware rescoring, docking, and short molecular dynamics refinements. Ensemble calibration, conformal prediction, and per-candidate confidence scoring will guide experimentalists toward the most promising candidates. Iterative active learning cycles will further optimize selection, reducing wasted assays and enabling efficient validation of lipid-modulating peptides (Fang et al., 2025; Di, 2015).

### 7.4 Precision peptide therapeutics and patient stratification

Future precision peptide programs will increasingly match peptide mechanisms with patient genotype, biomarker profiles, and population-scale sequence variability to maximize efficacy and safety (Rahman et al., 2016; Chen et al., 2020; Fritsch et al., 2020; Bak and Dai, 2015). Computational design will incorporate allele-specific binding simulations, population variability, and meta-learning adaptations for underrepresented subpopulations. Early developability, ADME, and immunogenicity filtering will reduce downstream attrition, while integrated computational-experimental-translational pipelines will enable rational prioritization of peptides most likely to succeed clinically. Investments in standardized datasets, uncertainty-aware models, and iterative experimental feedback are expected to substantially improve the translation of computational leads into safe and effective therapies.

## 8 Conclusion

The integration of computational methodologies into the discovery and optimization of bioactive peptides has fundamentally shifted the paradigm for developing novel lipid-modulating therapeutics. From the *in silico* screening of food-derived hydrolysates to the structure-guided design of orally bioavailable macrocyclic PCSK9 inhibitors, computational pipelines now compress discovery timelines and enable the rational engineering of stability, affinity, and specificity. Yet, the field remains constrained by the scarcity of high-quality, lipid-annotated peptide datasets, biases inherent in negative sampling, and the translational gap between robust preclinical efficacy and clinical validation. Addressing these challenges requires a shift from isolated predictor development toward integrated, iterative frameworks that combine deep learning-based functional prediction, physics-aware structural modeling, peptidomics-driven experimental filtering, and early consideration of developability and pharmacokinetics. Looking forward, the convergence of generative AI, population-scale genomics, and precision medicine paradigms holds promise for the design of patient-stratified peptide therapeutics capable of addressing residual cardiovascular risk. Ultimately, the successful translation of computational leads into clinical candidates will depend not only on algorithmic innovation, but on the rigorous, multi- orthogonal validation culture that bridges *in silico* inference and physiological reality.
